# Estrogens and Stem Cells in Thyroid Cancer

**DOI:** 10.3389/fendo.2014.00124

**Published:** 2014-07-25

**Authors:** Mariangela Zane, Veronica Catalano, Emanuela Scavo, Marco Bonanno, Maria Rosa Pelizzo, Matilde Todaro, Giorgio Stassi

**Affiliations:** ^1^Department of Surgical and Oncological Sciences, University of Palermo, Palermo, Italy; ^2^Department of Surgical, Oncological and Gastroenterological Sciences, University of Padua, Padua, Italy

**Keywords:** thyroid cancer, stem cells, cancer stem cells, estrogens, thyroid hormones, growth factors

## Abstract

Recent discoveries highlight the emerging role of estrogens in the initiation and progression of different malignancies through their interaction with stem cell (SC) compartment. Estrogens play a relevant role especially for those tumors bearing a gender disparity in incidence and aggressiveness, as occurs for most thyroid diseases. Although several experimental lines suggest that estrogens promote thyroid cell proliferation and invasion, their precise contribution in SC compartment still remains unclear. This review underlines the interplay between hormones and thyroid function, which could help to complete the puzzle of gender discrepancy in thyroid malignancies. Defining the association between estrogen receptors’ status and signaling pathways by which estrogens exert their effects on thyroid cells is a potential tool that provides important insights in pathogenetic mechanisms of thyroid tumors.

## Introduction

The endocrine system consists of a network of glands secreting hormones, which are chemical messengers that cooperate in growth, development, metabolism, and reproductive functions. The largest endocrine organ in the human body is the thyroid gland, whose function is the systemic metabolic regulation through thyroid hormones (THs) produced by follicular cells, and calcitonin produced by parafollicular cells. Different malignancy histotypes can arise from these cells: papillary (PTC), follicular (FTC), and anaplastic thyroid carcinomas (ATC) originate from follicular cells, while medullary thyroid carcinomas (MTC) derive from parafollicular cells (1). Notably, more than 95% of thyroid carcinomas (TCs) arise from follicular cells. These malignancies are indolent tumors treated by surgical resection with or without radioactive-iodine ablation since they maintain their distinct potential to concentrate Iodine. The loss of typical thyroid cell characteristics and functions, including expression of the thyroid-stimulating hormone (TSH) receptor (TSH-R), thyroglobulin (Tg), thyroid peroxidase (TPO), and sodium iodide symporter (NIS), defines the hallmark of ATCs, which are lethal malignancies with no effective therapy (1–3).

Besides genetic alterations in mitogen-activated protein kinase (MAPK), PI-3 kinase (PI3K), and TSH signaling pathways, thyroid carcinogenesis is fostered by the microenvironment, growth factors (GFs), and various hormones, including estrogens (4). Hormones can set off a cascade of signaling pathways, enhancing or contrasting specific effects triggered by other factors. Based on this *scenario*, the role of estrogens has been proposed in the pathogenesis of thyroid proliferative and neoplastic disorders. This hypothesis is supported by data regarding gender incidence, which reported a frequency of thyroid nodules about three to four times higher in women than in men with a peak rate occurring earlier in women (5, 6). Furthermore, the clarification of the estrogen-driven pathogenesis could be crucial in explaining why PTC constitutes the seventh most common cancer in the female gender (7, 8). An *in vivo* study reported that circulating estrogens are directly responsible for the increased female susceptibility to thyroid disease, through PI3K pathway activation and repressing p27 expression. The authors also observed a significant estrogen role in the transcriptional regulation of TPO, DUOX1, and NIS genes (9). Although several studies have demonstrated a direct action by estrogens on thyroid growth and function (7, 10–12), the precise mechanism underlying the proliferative and neoplastic disorders still remains undefined. In particular, it would be interesting to explore the role of hormones in TC initiation.

**Figure 1:**
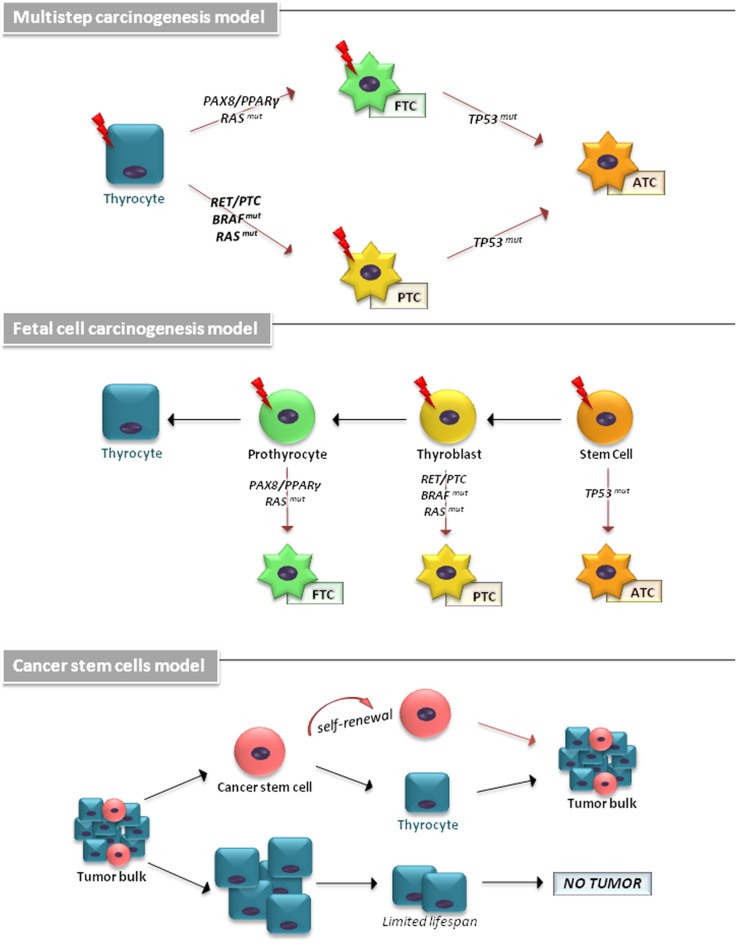
**The cellular origin of thyroid carcinomas is shown**. According to the multistep carcinogenesis model, TC originates from follicular cells as a consequence of multiple mutations accumulated throughout their life-span. Thyrocytes could give rise to PTC by RAS and BRAF mutations or RET/PTC and NTRK1 rearrangements and to FTC by point mutations of the RAS gene and PAX8/PPARγ rearrangement. ATC derive from PTC and FTC after deregulation of the p53 and the Wnt/β-catenin pathway. In fetal cell carcinogenesis model, three types of fetal thyroid cells were proposed to generate different forms of thyroid cancer. Fetal thyroid stem cells, characterized by expression of the oncofetal fibronectin (OF), generate ATC, thyroblasts, which express OF and the differentiation marker Tg, are proposed to be the cellular origin of PTC. The more differentiated prothyrocytes, expressing Tg, give rise to FTC. The cancer stem cells model proposes TC as an SC disease. The accumulation of mutations in differentiated thyrocytes leads to their transformation. A subset of these cells may (in more aggressive tumor types) dedifferentiate and assume CSC characteristics.

The cellular origin of TCs has been explained by different models (Figure 1). The multistep carcinogenesis model predicts that TC originates from follicular cells as a consequence of multiple mutations accumulated throughout their life-span. These events are characterized by a dedifferentiation process with a marked epithelial-to-mesenchymal transition (EMT), in which well-differentiated TC cells transform into a more undifferentiated phenotype (1). The fetal cell carcinogenesis model hypothesizes that TC cells would be generated by transforming three types of fetal thyroid cells, stem cells (SCs), thyroblasts, and prothyrocytes, which result in ATC, PTC, and FTC, respectively (13, 14). The heterogeneity of tumor bulk had led to a cancer stem cells (CSCs) model to propose TC as an SC disease. The growing body of experimental evidence has revealed that an accumulation of genetic abnormalities in tissue-resident SCs or in their more committed progenies, concomitant with the niche epigenetic alterations, result in their malignant transformation (15, 16).

The “cell-of-origin” concept explains how a normal cell acquires the first alteration able to trigger tumor initiation (tumor-initiating cells, TICs) (17). Wnt pathway plays a crucial role in SC/progenitor compartment maintenance, and has been described in several tumors, including TC, resulting in nuclear β-catenin-induced proliferation (18–20).

In this review, the most current findings supporting the carcinogenesis effects of estrogens and THs will be addressed. A special emphasis will be given to the role of exogenous and endogenous GFs affecting thyroid proliferative pathways in SC compartment.

## Estrogens

As recently published by Morrison’s research group, estrogens are involved in increasing hematopoietic SC self-renewal in female subjects and more specifically during pregnancy (21). It is likely that normal and tumor thyroid tissues, which express estrogen receptors (ER), could be subject to the same mechanism of estrogen action (10, 22–24).

Involved in cellular processes such as growth, cell motility, and apoptosis, in reproductive tissues and other organs, including endocrine glands, estrogens are mainly produced by the adrenal cortex and ovary, but also by the thyroid (25, 26). They are present in women and men with a notable increase in women at reproductive age. The three principal estrogens, estrone (E1), estradiol (E2), and estriol (E3), are processed in metabolites with different estrogenic abilities, which create a different risk in developing cancer (27–29).

**Figure 2:**
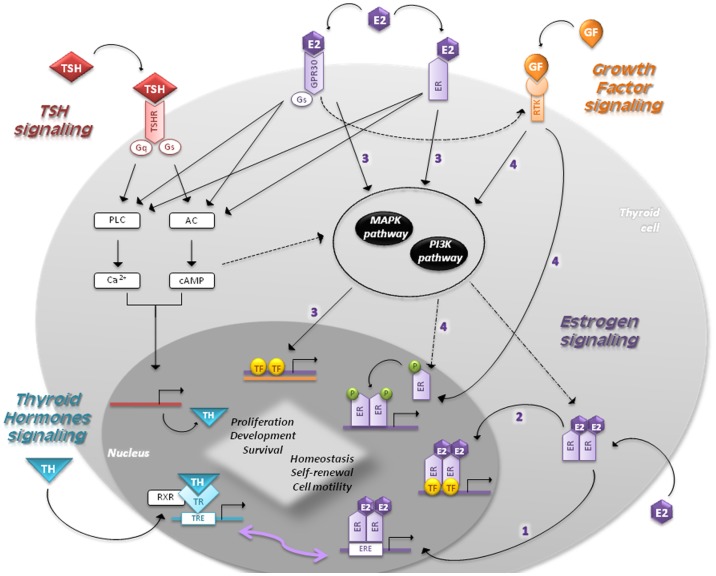
**Signaling pathways in follicular cells are shown**. The main regulators of thyroid proliferation and function act through TSH signaling and GF pathway. THs control the secretion of TSH, which binds to TSH-R and induces the coupling of G-proteins stimulating AC and PLC. TSH also acts via PI3K pathway. GFs act via MAPK and PI3K pathways regulating the expression of genes involved in survival, cell cycle progression, and proliferation. Estrogens regulate proliferation, cell motility, differentiation, and apoptosis through four different mechanisms: (1) *genomic (or classical) estrogen-signaling*: E2–ER complex translocates into the nucleus, where it binds to ERE-sequences; (2) *ERE-independent genomic actions (TFs cross-talk)*: genes lacking in ERE-sequences are activated by other TFs in the nucleus through protein–protein interactions; (3) *non-genomic (or membrane-initiated) estrogen-signaling*: E2 activation of plasma membrane-associated ER and GPR30 trigger the activation of MAPK and PI3K pathways and/or increases the Ca^2+^ levels; (4) *ligand-independent signaling*: in absence of E2, GFs can stimulate ERs directly or indirectly through MAPK and/or PI3K pathways. THs play a critical role in development and homeostasis. Nuclear TRs activate gene expression by binding to RXR, which in turn bind to TRE-sequences. Given that EREs share a similar nucleotide sequence with TREs, ERs and TRs can interact and regulate several transcriptional responses. The cross-talk between genomic and non-genomic pathways and other integrative signaling lead to a synergic cell response.

Estradiol is the most potent estrogen since it has the highest affinity to its receptors. Estrogens perform their function by binding to ER alpha and beta (ER-α, ER-β), and a transmembrane intracellular non-classical ER G-protein-coupled receptor 30 (GPR30) (Figure 2). ER-α and ER-β are soluble intracellular nuclear receptors, belonging to a ligand-dependent nuclear receptor superfamily of transcription factors (TFs) (25, 26). ER-α is the key factor of E2-induced proliferation with an anti-apoptosis effect. In females of reproductive age, ER-α levels are higher in PTC compared to nodular goiter patients, showing a positive correlation between ER-α and Ki-67 expression levels. In contrast, ER-β is associated with apoptosis and growth inhibition, providing a negative correlation with mutant P53 (30). PPARγ also interacts with ER-α inhibiting each other, and with ER-β enhancing their inhibitory effect on cell proliferation and migration (31). In light of this, the ER-α/ER-β *ratio* could be helpful to elucidate the TC pathophysiology (25, 32).

The interaction between estrogens and ERs signals through different pathways:

- Genomic (or classical) estrogen-signaling: after accessing the cell through passive diffusion, E2 binds to ER, which changes its conformation and homo- or heterodimerizes (E2–ER). This complex translocates into the nucleus, where it binds to the 15-bp palindromic estrogen response element (ERE) located in the regulatory regions of target genes. This interaction leads to a co-activators recruitment, which in turn allows expression of genes involved in proliferation (33, 34).
- Estrogen response element-independent genomic actions (TFs cross-talk): ERE-lacking genes can be activated by modulating other TFs through protein–protein interactions. This molecular mechanism induces chromatin remodeling, histone unwinding, and interaction with the basal transcription machinery complex (35–37).
- Non-genomic (or membrane-initiated) estrogen-signaling: E2 activation of plasma membrane-associated ER and GPR30 promotes the MAPK and PI3K signaling pathways and/or increases the Ca^2+^ levels (10, 38–40). They can also activate G-proteins resulting in cAMP production, similar to TSH signaling in thyrocytes, and assist the activation of metalloproteinases (MMPs) and the GF pathway (5).
- Ligand-independent signaling: in absence of E2, GFs can stimulate ERs directly or indirectly through MAPK and/or PI3K pathways (41).

The cross-talk between genomic and non-genomic pathways, as well as the integrative signaling by E2 in different cell compartments, leads to a synergy that provides plasticity in cell response. Estrogens dispatch their proliferative role also by increasing T_3_ levels and stimulating the iodine-uptake and TPO activity (42).

Furlanetto et al. (43) reported that E2 increases proliferation of thyroid cells down-regulating NIS. These data underline the pivotal role of estrogens in the SC compartment maintenance. In normal and tumor thyroid cell lines, Rajoria et al. documented that E2 is associated with increased proliferation, adhesion, invasion, and migration via β-catenin (7) and MMP-9 modulation (44). Likewise, E-cadherin down-regulation and β-catenin translocation sustain the metastatic activity of TC cells (24). These results confirmed the findings by Kouzmenko et al., which reported the first evidence of cross-talk between estrogens and Wnt pathways through functional interaction of β-catenin with ER-α (45).

Xu et al. (8) analyzed whether differentiated and SC/progenitors could be target of estrogen action in thyroid. SCs isolated from goiter tissue enhanced their sphere-forming ability in presence of E2. Moreover, thyroid-sphere cells showed ER-α mRNA levels eight times higher than those of more differentiated thyrocytes. This suggests the gender discrepancy in TC incidence and a difference in terms of aggressiveness and survival.

## Thyroid Hormones

Thyroid hormones control the secretion of thyrotropin-releasing hormone (TRH) from the hypothalamus and TSH from the anterior pituitary through negative feedback loops (1). Thyroid homeostasis and function are regulated by a concert of signals accumulated from TSH and GF pathways. TSH binds to TSH-R and induces the coupling of different G-proteins, stimulating adenylate cyclase (AC) and phospholipase C (PLC) (Figure 2). This promotes iodide uptake and TG, TPO, and NIS expression, producing thyroxine (T_4_) and triiodothyronine (T_3_) (19, 46). On the contrary, intracellular Ca^2+^ and PLC regulate iodine release, H_2_O_2_ production, and Tg iodination (47, 48). Although cAMP is the main mediator of TSH stimulation in thyroid cell growth, TSH via PI3K increases cyclin E levels leading to cell cycle progression (49, 50). TSH-R is also associated to the MAPK pathway through its desensitization and internalization apparatus (51).

Gain-of-function mutations in TSH-R or Gs genes result in increased cAMP accumulation and TSH-independent proliferation, which account for hyperfunctioning nodules in patients with multinodular goiters (52, 53). These alterations result insufficient for the malignant transformation of thyroid cells (54, 55). Hence, it is likely that other factors intervene in the SC compartment, which is assumed to be the target of neoplastic transformation. Alterations of the Wnt pathway effectors are involved in cancer initiation and progression (56). In particular, TSH-mediated Wnt-1 over-expression and GSK-3β inhibition promote thyroid cell proliferation (57, 58).

Thyroid hormones play a critical role in the tissue development and homeostasis by direct transcriptional regulation or modulation of different pathways (59). Although T_4_ is the predominant hormone produced by the thyroid, T_3_ is the active form that mediates gene regulation binding with a higher affinity to thyroid receptors (TRs) (60). Nuclear TRs activate gene expression by binding with the retinoid X receptors (RXRs) to TH response elements (TRE), located on the promoters of target genes (Figure 2) (61). Given that EREs share a similar nucleotide sequence with TREs, ERs and TRs can interact and regulate several transcriptional responses to environmental *stimuli* (5). Interestingly, ERE can act as a peroxisome proliferator responsive elements (PPRE), binding PPARγ/RXR. It can henceforth inhibit ER transactivation through a competition for ERE binding (62). In line with this cross-interaction, the proliferative effect of estrogens on human NPA-87-1 PTC cell line is TSH-independent (63). Lima et al. demonstrated a more direct proliferative effect since E2 administration to prepubertal and adult rats enhances thyroid weight without significant changes in T_3_, T_4_, and TSH hematopoietic levels (42).

Recent studies in human cancers and mouse models provide strong evidence that the loss of TRs function contributes to cancer initiation and progression (64). While the TRα1 trigger directly promotes transcription of CTNNB1 (65, 66), the effect generated by the TRα2 stimulation in SC compartment is still unknown. Cross-talk between THs-TRα1 and Wnt pathway has been confirmed by the up-regulation of several SC markers (67). Furthermore, it was reported that aberrant nuclear localization of β-catenin-induced by CTNNB1 mutations contributes to the progression of ATCs (68). Data reported by Todaro et al. showed that E-cadherin down-regulation together with β-catenin activation confers an invasive capacity and higher metastatic rate to thyroid CSCs (18).

## Growth Factors

In thyroid, GFs exert their proliferative effects by inducing the RTK dimerization that activates the downstream PI3K pathway and the MAPK cascade via G-proteins (Figure 2). Alterations in genes involved in the MAPK pathway led to its constitutive activation, which represents a typical feature of TC (1). In particular, mutations in RET and NTRK and alterations in RAS and BRAF intracellular signal-transducers are clearly implicated in PTC pathogenesis (69). RAS point mutations and PAX8/PPARγ rearrangement have been frequently implicated in FTC pathogenesis (70, 71). The inactivation of *RASAL1* (encoding a RAS GTPase-activating protein) by hypermethylation and mutations provides a new genetic background for FTCs and ATCs (72). Besides nuclear β-catenin accumulation and p53 inactivation, oncogenic activation of MAPK and PI3K/Akt/Foxo3a are frequently found in ATCs (2, 73, 74). The acquisition of a TERT promoter mutation was recently associated with clinical–pathological aggressiveness in FTCs and BRAF mutation-positive PTCs (72, 75).

The mesenchymal tissue is involved in thyroid development being that it releases Pro-epidermal growth factor (EGF) and basic fibroblast growth factor-2 (FGF-2), promoting cell proliferation and repressing differentiation (76, 77). Estrogens play a pivotal role in this context by inducing the production of EGF and other TFs, such as TGF-α (5).

After EGF binding, RTKs of the ErbB family (EGFR/ErbB1, ErbB2, ErbB3, and ErbB4) achieve activation through the arrangement in homo- and/or heterodimeric complexes (78, 79). In thyroid, TSH increases the expression of EGFRs that in turn promote the EGF mitogenic effect and contribute to gland homeostasis. The combination of specific EGFRs regulates the stimulation intensity, inducing transformation. Indeed, an increased expression of EGFRs in TCs compared to normal tissue has been reported (80). EGFR/ErbB1 over-expression and its constitutive phosphorylation have been observed on ATC samples and cell lines (81). Their expression has been retrieved in 90% of the PTC samples examined by Song (82). In combination with the repression of VEGF, EGF inhibitors could be a promising therapy for ATCs as demonstrated by *in vitro* studies (83, 84). EGF is also supplemented in the serum-free culture medium, which is used to isolate SCs and CSCs *in vitro* (18, 85–90).

Similarly, the cell response to FGF is regulated by FGF RTKs (FGFRs 1–4). FGF-2 exerts autocrine and paracrine stimulatory effects on thyroid growth, since the basement membrane of thyrocytes is able to produce FGF itself. FGF is also used *in vitro* for the maintenance of SC niche (18, 91); in particular, it could have an inhibitory effect on thyroid function through cAMP inhibition and TSH’s activity weakening (79). In TC, increased FGF-2 levels and FGFR2 over-expression are critical in tumor progression and neovascularization (92, 93). Therefore, the differential expression in normal and malignant conditions could make this receptor a potential diagnostic marker for TCs (94).

Growth factors also affect development and metabolic processes through insulin-like growth factor (IGF). After binding of their ligands, IGF receptors (IGF-Rs) autophosphorylate their intracellular domain and activate the MAPK and PI3K cascade (95). Consistently, IGF enhances the TSH mitogenic effect on follicular cells (96); on the other hand, it also cooperates with FGF-2 in establishing and maintaining the SC niche *in vitro* (96). Indeed, IGF pathway effectors are over-expressed in CSCs: IGFR2 is involved in an autocrine loop that sustains SC renewal, and IGF increases the expression of Oct-4 and Nanog when added to the culture medium (87, 97, 98).

## Estrogen-Growth Factors Interacting Proteins

Recently, there has been a focus on importance of the ER-GFs interacting proteins on cancer cell proliferation and invasivity. An example is mediator of ERbB2-driven cell motility (MEMO), which enhances ER-α extra-nuclear functions through the interaction with IGFR1 and ERbB2, activating MAPK and PI3K signaling (99).

## Concluding Remarks

Since the theory of fetal carcinogenesis has initially been postulated, thyroid CSCs have been studied for their potential role as TICs. It has been hypothesized that various factors could be involved in the malignant transformation, such as aberrant molecular events converging to RTK, MAPK, and PI3K pathway activation. Besides the oncogenes contribution, it is likely that a network of various hormones and GFs could maintain the SC niche and enhance the proliferation of progenitors sustaining tumor bulk growth. Indeed, recent studies demonstrate that sexual hormones could exert a supportive role in the propagation of SCs and progenitors, as suggested by the cross-talk between estrogen-signaling and Wnt pathway. Furthermore, the latter pathway has also been observed interacting with THs in SC compartment and so accelerating tumorigenic processes. This mechanism could be benefited by the interaction between different cascades, which enhances or contrasts specific cellular response in tumor conditions. In conclusion, an in-depth study on the concert between estrogens, THs, and GFs could be helpful to elucidate hormones-driven thyroid carcinogenesis. Gaining more insight into this interaction could also explain the gender imbalance in tumor incidence for the purpose of identifying a more targeted approach in TC therapy.

## Conflict of Interest Statement

The authors declare that the research was conducted in the absence of any commercial or financial relationships that could be construed as a potential conflict of interest.
